# Current and Future Needs for Human Resources for Ethiopia’s National Health Information System: Survey and Forecasting Study

**DOI:** 10.2196/28965

**Published:** 2022-04-12

**Authors:** Binyam Tilahun, Berhanu F Endehabtu, Kassahun D Gashu, Zeleke A Mekonnen, Netsanet Animut, Hiwot Belay, Wubshet Denboba, Hibret Alemu, Mesoud Mohammed, Biruk Abate

**Affiliations:** 1 Department of Health Informatics Institute of Public Health, College of Medicine and Health Sciences University of Gondar Gondar Ethiopia; 2 Health Systems Directorate Ministry of Health Addis Ababa Ethiopia; 3 Data Use Partnership Project John Snow, Inc (JSI) Addis Ababa Ethiopia; 4 Policy, Planning, Monitoring and Evaluation Directorate Ministry of Health Addis Ababa Ethiopia

**Keywords:** forecasting, human resources, health information system, workforce, Ethiopia, health informatics, healthcare professionals

## Abstract

**Background:**

Strengthening the national health information system is one of Ethiopia’s priority transformation agendas. A well-trained and competent workforce is the essential ingredient to a strong health information system. However, this workforce has neither been quantified nor characterized well, and there is no roadmap of required human resources to enhance the national health information system.

**Objective:**

We aimed to determine the current state of the health information system workforce and to forecast the human resources needed for the health information system by 2030.

**Methods:**

We conducted a survey to estimate the current number of individuals employed in the health information system unit and the turnover rate. Document review and key-informant interviews were used to collect current human resources and available health information system position data from 110 institutions, including the Ministry of Health, federal agencies, regional health bureaus, zonal health departments, district health offices, and health facilities. The Delphi technique was used to forecast human resources required for the health information system in the next ten years: 3 rounds of workshops with experts from the Ministry of Health, universities, agencies, and regional health bureaus were held. In the first expert meeting, we set criteria, which was followed by expert suggestions and feedback.

**Results:**

As of April 2020, there were 10,344 health information system professionals working in the governmental health system. Nearly 95% (20/21) of district health offices and 86.7% (26/30) of health centers reported that the current number of health information system positions was inadequate. In the period from June 2015 to June 2019, health information technicians had high turnover (48/244, 19.7%) at all levels of the health system. In the next ten years, we estimate that 50,656 health information system professionals will be needed to effectively implement the Ethiopia's national health information system.

**Conclusions:**

Current health information system–related staffing levels were found to be inadequate. To meet the estimated need of 50,656 multidisciplinary health information system professionals by 2030, the Ministry of Health and regional health bureaus, in collaboration with partners and academic institutions, need to work on retaining existing and training additional health information system professionals.

## Introduction

A health information system integrates data generation, compilation, analysis, and synthesis, as well as the reporting and use of the information in evidence-based decision-making. The system can be a hybrid of both electronic and paper-based data collection from health institutions and other related sectors and ensures overall data quality. A strong health information system is central to achieving better health outcomes and is the backbone of a strong health system [1].

Strengthening the Ethiopia’s health information system, so that it can produce quality and actionable health data, is critical for planning, mobilizing resources, establishing policies, monitoring health health-related activities and performance [2]. The Ministry of Health of Ethiopia has set strategies to establish a well-functioning health information system with the aim to improve evidence-based decision-making through enhanced partnership, harmonization, and alignment, including the integration of projects and programs at the point of health service delivery [3].

An effective health system is a function of multiple factors. Among the 6 building blocks of its framework for health systems, the World Health Organization includes *human resources* [4,5]. Human resources consume the most financial resources in the health sector; human resources also influence the efficiency and effectiveness of other health system building blocks. Although health sector–related human resources have been persistently neglected for years, currently, more attention is being paid to human resources which is considered, both by the global health community and national governments, to be a critical component in improving health outcome [4,6,7]. An adequate amount and mix of skilled human resources are essential at all levels of the health system. The level and mix of human resources required depend on the goals and objectives of a specific health system [6,8-10].

Globally, countries have been challenged by health care workforce shortages and uneven distribution of skills and professions [11]. The problem is worse in low- and middle-income countries. Ethiopia is one of the countries listed by the World Health Organization as having a health workforce crisis [12]. The implementation of a health information system in Ethiopia has been challenged with shortages of skilled human resources, a poor distribution of professions, and high attrition rates at all levels of the health system for activities that include the collection of reliable and complete information, data analysis and interpretation, and effective use of data for planning and decision-making [11,13].

Even though the country has set strategies to strengthen the health information system, the need for health information system human resources has not been quantified or characterized. Shortages, low skill, and uneven distributions in the workforce will be aggravated with time due to factors such as an expansion of health facilities, population growth, new technology in the health system, individual levels of performance, turnover rates, and health policy [14,15]. Higher education institutions in Ethiopia have shown interest in health information system workforce development; however, there is no national roadmap. Currently, some health science colleges and public universities have opened Diploma and Bachelor of Science Health Information System programs. In addition, some universities have started postgraduate-level Health Informatics programs.

Determining the performance level of the current workforce will help to identify the skills and knowledge required by those jobs and the human resources needed [16]—forecasting considers existing and future human resources–related demand and plans at all levels of the health system. Estimating future health information system human resources is also expected to help in addressing the issues of employing, educating, allocating, retaining, motivating, and managing the workforce, to ultimately improve the health information system.

Delphi is one technique used [17-19] for forecasting requirements. It is particularly helpful when the area of investigation does not allow for the use of analytical techniques but is suitable for expert opinion. The technique helps to facilitate a structured and systematic group communication process [19]. The Delphi technique allows efficient use of experts’ diverse knowledge and experiences for the prediction of future human resources demand, despite limitations such as the inability to take into account complex factors that cannot always be predicted accurately by experts [18,20-22].

We aimed to determine the current status of the health information system workforce and forecast the human resources required for the health information system by 2030.

## Methods

### Ethics

The study protocol was reviewed and approved by ethical review board of the University of Gondar (number RCS/768/19). Informed consent was obtained from each study participant. A letter of permission was also obtained from each regional health bureau. The names of participants and other personal identifiers were not collected in the study.

### Study Design

From June 2019 to April 2020, we conducted a national-level assessment of the health information system in Ethiopia in all regions and 2 city administrations to estimate the current amount of human resources and the turnover rate. We collected data from a total of 110 organizations: the Ministry of Health, 4 federal agencies, 11 regional health bureaus, 18 zonal health departments, 21 district health offices, 5 referral hospitals, 12 general hospitals, 8 primary hospitals, and 30 health centers. A total of 278 institution heads, planning department heads, and health management information system heads were interviewed.

We asked each facility and organization to collect and send information on required human resources to the ministry of health officials, and then, these data were used to forecast national human resources requirements. We use the Delphi technique—3 rounds of workshops were held with experts from the Ministry of Health, universities, agencies, and regional health bureaus.

### Operational Definition

We defined *health information system professionals* as professionals in the fields *health informatics*, *health system*, *health management*, *computer science and information technology*, *monitoring and evaluation*, and *biostatistics* and other professionals who are directly involved in technical health information system activities such as planning, monitoring and evaluation, system analysis and design, and system administration.

### Data Collection

A team (2 to 5 data collectors) was assigned in each region. Two days of training were provided to data collectors. Data collectors were health informatics, health service management, monitoring and evaluation, biostatistics, and health economics professionals. Health system experts from the Policy Plan Monitoring and Evaluation Directorate of the Ministry of Health participated as supervisors. Interview and document review methods were used to collect data.

### Delphi Process

Experts and policy makers from the Ministry of Health and experts from universities, federal agencies, regional health bureaus, and other stakeholders were involved in each round. A total of 13 experts were involved in the forecasting process.

Data collected from the facilities and organizations were used to identify and prioritize major factors that could affect estimates for human resources for health information system. A total of 14 criteria were listed for rating, of which future expansion plans, the existing amount of human resources for health information system, the number of organizations, the number of standard health information system positions, and the turnover (or attrition) rate were selected based on ratings to be used for forecasting human resources requirements by 2030.

For the validation process, variables such as current number of health information system positions, currently available health information system workforce, and human resources required for health information system had no variation across facilities. These data did not differ across facilities and were found to be normally distributed (through visual assessment of the histograms).

## Results

### Current Human Resources

#### Overview

A total of 10,344 health information system workers were found to be actively working at different levels of the health system as of April 2020. Approximately half (5685/10,344, 55.0%) were diploma-level health information technicians. Staff had an average of 3 years of professional work experience. At the Ministry of Health, 24 employees worked in health information system–related positions (health science: 6/24, 25%; computer science: 6/24 (25%); health informatics: 4/24, 16.7%).

In regional health bureaus, there were 160 health information system–related professionals (health science: 48/160, 30%; computer science: 48/160, 30%; health informatics: 20/160, 12.5%). In all district health offices, a total of 45 workers were assigned to health information system–related activities, of which 19 (42%) were diploma-level health information technicians. Between April 2015 and April 2020, a total of 218 employees left their jobs; diploma-level health information technicians had the highest turnover rate (48/244, 19.7%) at the national level.

We found that health informatics, information technology, health information technician, environmental health, monitoring and evaluation, public health officer, health economics, statistics, Master of Public Health, business management, management, nursing, health officer and computer science graduates were currently working on health information system–related activities at each level of the health system.

#### Health Information System–Related Positions

The Ministry of Health had 13 health information system positions; however, it was reported that the current number of positions was not adequate, and it was suggested that the number be increased to 40. Federal agencies had an average of 14 positions. Regional health bureaus had an average of 6 positions, and 10 of 11 regional health bureaus reported not having an adequate number of positions; it was suggested that the number of positions be increased to 8 per region. The average number of health information system positions for zonal health department was 2. Fifteen zonal health offices and departments reported having an inadequate number of health information system positions; it was suggested that the number of positions be increased to an average of 4. District health offices had an average of 2 positions. Of those assessed, only 68.2% (30/44) had budgeted for health information system position; 95% (20/21) reported that current number of positions was not adequate and suggested that that the number of positions be increased to 5 per district. Health centers had an average of 1 health information system position; the majority (26/30, 86.7%) suggested that the number of positions be increased to 3 per health center. Three-quarters (6/8, 75%) of the primary hospitals reported not having an adequate number of positions and suggested having 5 positions per primary hospital. General hospitals had an average of 4 health information system positions, and the majority (9/12, 75%) reported that the number of positions was inadequate. Specialized or referral comprehensive hospitals had an average of 6 health information system positions, of which 80% (4/5) were budgeted. The majority (4/5, 80%) of comprehensive specialized or referral hospitals reported that the existing number of health information system positions was inadequate and suggested having 19 positions per specialized or referral hospital (Table S1 in Multimedia Appendix 1).

### Estimated Human Resources Needed by 2030

#### Overview

The Delphi technique was implemented in 2 weeks, and 8 criteria were used to forecast human resources requirements by 2030: future expansion plan, current amount of human resources, current number of organizations, number of standard health information system positions, eHealth initiatives, strategic plan, budget for health, and turnover (or attrition) rate (Table S4 in Multimedia Appendix 1).

#### At the Federal Level (the Ministry of Health and its Agencies)

Based on the criteria prioritized above, it was estimated that 90 positions will be needed by the Ministry of Health (Master of Health Informatics: 27/90, 30%; Master of Monitoring and Evaluation: 22/90, 24.4%; Master of Information Technology or Computer Science: 21/90, 23.3%). Moreover, it was estimated that 105 positions will be needed for all federal agencies after considering all potential factors affecting human resources development—on average, each federal agency will require 15 employees to work on health information system–related activities—and the majority would need to have a Master of Health Informatics degree (28/105, 26.7%) (Table S7 and S8 in Multimedia Appendix 1).

#### At the Regional Level (Regional Health Bureaus and Regional Agencies)

Approximately 276 health information system positions will be needed at regional health bureaus, which translates to 25 per region. Most regional health bureaus would require professionals with a Master of Health Informatics degree (55/276, 20%), followed by those with Bachelor of Science degrees in Computer Science or Information Technology professionals (53/276, 19.2%). In all regional agencies, 63 positions will be required—regional agencies will require an average of 9 employees to work on health information system–related activities (Tables S9 and S10 in Multimedia Appendix 1).

#### Zonal Health Departments and District Health Office

During the survey, 172 health workers were working on health information system–related activities at the zonal health department level. The forecast estimate of human resources was 624 (Table S11 in Multimedia Appendix 1). During the survey in all district health offices, 1428 health workers were working on health information system related activities. A total of 3060 professionals will be needed in district health offices by 2030 (Master of Health Informatics: 1020/3060, 33%; Bachelor of Science in Health Informatics: 1020/3060, 33%; Bachelor of Science in Information Technology or Computer Science: 1020/3060, 33%) (Table S12 in Multimedia Appendix 1).

#### Primary, General, Comprehensive, and Specialized Hospitals

A total of 1419 professionals will be needed in comprehensive specialized hospitals by 2030. More than half (817/1419, 57.6%) will be diploma-level health information technicians (Table S13 in Multimedia Appendix 1). There are currently 79 general hospitals, and given the expansion plan, the number of general hospitals will increase to 150. For general hospitals, 2100 professionals will be needed, given that medical record units will be staffed with 7 health information technicians per hospital (Table S14 in Multimedia Appendix 1). There will be a total of 505 primary hospitals by 2030 (211 currently existing and 294 planned), which will require 4545 health information system professionals, with an average of 9 per hospital—5 diploma-level health information technicians e for the medical record unit, and the rest will be for data management—to implement telemedicine and harness information technology development (Table S15 in Multimedia Appendix 1).

#### Health Centers

Based on the assessment, there will be a total of 4586 health centers by the year 2030. There are currently 3591 health centers, and 995 will be constructed according to the expansion plan. A total of 27,516 professionals (6 per health center) will be required to support the implementation of electronic community health information systems at the health post level and eHealth initiatives in the health system (Table S16 in Multimedia Appendix 1).

#### Private Health Facilities

The health information system in Ethiopia has been engaging the private health facilities to follow the national health information system guidelines. Private health facilities could demand human resources for health information system in the future. At least 1 diploma-level health information technician for health information system and 1 diploma-level information technology employee for information communication technology support will be required at each private health facility (Table S17 in Multimedia Appendix 1).

#### Universities and Health Science Colleges

Universities educate and deploy the health professionals that work for the health system, and accordingly, need to have competent staff to train the workforce that will work on health information system–related activities. The experts suggested that 10 of the 55 universities will have health informatics departments by 2030, each requiring 48 professionals (Table S18 in Multimedia Appendix 1). Health science colleges regularly train health information technicians at the diploma level. It was estimated that 27 health science colleges, with 12 professionals each, will be required (Table S19 in Multimedia Appendix 1).

#### Total Estimated Workforce

A total of 50,656 of health information system employees will be needed by 2030 at the national level. More than half (27,516/50,656, 54.3%) of the workforce will be needed by health centers (Table S5 in Multimedia Appendix 1).

## Discussion

The study has shown that the current number of health information system–related positions and the existing workforce were found to be inadequate. There was high turnover in the health information system–related workforce. It was forecast that 50,656 multidisciplinary health information system professionals will be needed nationally by 2030.

From the assessment, we found that approximately 10,344 employees were currently working on health information system–related activities and many district health offices (20/21, 95%) and health centers (26/30, 86.7%) reported that the number of health information system positions was inadequate. This finding indicates that current human resources are inadequate to achieve the national agenda. The level of skill mix was unbalanced. The majority of the health information system workforce currently working on health information system–related activities were diploma-level health information technicians. The correct amount and mix of the workforce is needed to establish well-organized health system [23,24].

We found that there were professionals from fields with no or little relation to health information system activities, such as environmental health, monitoring and evaluation, public health officer, health economics, statistics, Master of Public Health, business management, management, nurse, health officer, and computer science, employed in the health information system. Misplacement of employees in the workforce affects the effectiveness and efficiency of the organization [23]. Hence, every organization must have the right profession with the right knowledge and skill at the right place.

Our findings showed that there was high turnover in the health information system workforce; however, the reason for the turnover and its impact were not assessed in this study. Higher turnover is costly and associated with poor health service outcomes [25]. Health information system tasks, such as managing health care data and infrastructure, are complex and require experienced and well-trained professionals. Well-trained and experienced staff turnover is costly, mainly due to the need to train new employees, and compromises health system performance and the quality of services [8,25,26].

Educating and recruiting alone will not alleviate the workforce shortage. Assessment of the factors related to job satisfaction and retention is critical [27]. We found that diploma holders were the majority (48/244, 19.7%) of those who left their positions. This may be as a result of a lack of professional development opportunities. Until 2018, there was no academic program to upgrade their qualifications, and individuals with diplomas would have to upgrade by do a Bachelor of Science degree in nursing or other health science fields. The University of Gondar now has a post basic degree program in health informatics. There is evidence suggesting that improving the literacy of health workforce increases the retention rate [28]. Therefore, the Ministry of Health should design a strategy to provide scholarship opportunities and other job satisfaction schemes to minimize turnover.

The need for long-term forecasting of human resources for a health system in uncertainty and at the national level is a complex process—different variables must be considered in estimating the amount, skills, and professional mix of health personnel for meeting health system needs, which is information that policy and decision-makers need [29].

Different methods can be used to forecast human resources needs [29-31]. We used the Delphi method. The health information system workforce needed by the end of 2030 will be greater than 50,000. This indicates that the Ethiopia’s shortage in health information system human resources is critical. Therefore, the Ministry of Health and the Ministry of Education should work together to ensure that there will be an adequate number of health information system graduates. There are some limitations to this study. First, in collecting data, we used the bottom-up managerial judgment approach and did not consider the final opinion of the bottom managers. Second, we did not collect all facility and administrative office managers' opinions; therefore, forecasts could be higher or lower than the actual need. Third, we assessed the magnitude of health information system staff turnover but we did not address the reasons.

This study has shown that the current health information system workforce is currently inadequate and will be inadequate for future needs. We estimated that approximately 50,656 human resources for health information system will be needed by 2030. Hence, the Ministry of Health and regional health bureaus, in collaboration with health information system and health system strengthening partners, need to train more health information system professionals and develop mechanisms to retain experienced professionals in the health system. Strong collaborations are also needed with universities to ensure the right skills are included within health information system curricula.

## Appendix

Multimedia Appendix 1 Supplementary material.

## Data Availability

The data sets are available upon request from the corresponding author.
