# Supplementary material for: Current and Future Needs for Human Resources for Ethiopia’s National Health Information System: Survey and Forecasting Study
Source: JMIR Med Educ. 2022 Apr 12;8(2):e28965. doi: 10.2196/28965 (PMC9044145; doi:10.2196/28965)
Supplement: Multimedia Appendix 1 [file mededu_v8i2e28965_app1.docx]

**Appendix 1**

Table 1: Existing and suggested HIS positions at each level of the health system

| **Type of organization** | **Sample size** | **No. of current HIS positions in organization** | **Average no. of current HIS positions in organization** | **Total number of budgeted HIS positions in organization** | **Proportion of budgeted HIS positions in the organization** | **Proportion of organizations with adequate HIS positions** | **Total number of suggested HIS positions** | **Average no. of suggested HIS positions*** |
| --- | --- | --- | --- | --- | --- | --- | --- | --- |
| MOH | 1 | 16 | 16 | 1 | 6.3% | 0%) (0/1) | 40 | 40 |
| Federal Agencies | 4 | 57 | 14 | 48 | 84.2% | 25%(1/4) | 80 | 27 |
| RHB | 11 | 64 | 6 | 39 | 60.9% | 9% (1/11) | 87 | 8 |
| Zonal office | 18 | 38 | 2 | 28 | 73.7% | 16.7% (3/18) | 63 | 4 |
| District office | 21 | 44 | 2 | 30 | 68.2% | 5% (1/21) | 98 | 5 |
| Health Center | 30 | 35 | 1 | 30 | 85.7% | 13.3% (4/30) | 76 | 3 |
| Primary hospital | 8 | 17 | 2 | 13 | 76.5% | 25% (2/8) | 29 | 5 |
| General hospital | 12 | 50 | 4 | 31 | 62% | 25% (3/12) | 66 | 7 |
| Specialized/Referral hospital | 5 | 31 | 6 | 25 | 80.6% | 20% (1/5) | 75 | 19 |

**Visited federal agencies include: HAPCO, EPHI, Blood Bank and FMHACA*

**Average no. of suggested HIS positions was calculated by those who reported inadequate HIS positions*

Table 2: National HIS manpower working at each level of the health system during the survey

| **Type of organization** | **Health Informatics** | | | | **M & E** | **Information Technology** | | | **Health science** | | | **Statistics** | | **Computer Science** | | **Others** | **Total** | **Average** |
| --- | --- | --- | --- | --- | --- | --- | --- | --- | --- | --- | --- | --- | --- | --- | --- | --- | --- | --- |
|  | **Diploma** | **BSc** | **Masters** | **PhD** | **Masters** | **Diploma** | **BSc** | **Masters** | **Diploma** | **BSc** | **Masters** | **BSc** | **Masters** | **BSc** | **Masters** |  |  |  |
| **MOH** |  | 1 | 3 | 0 | 4 | 0 | 0 | 0 | 0 | 2 | 4 | 0 | 0 | 4 | 2 | 4 | **24** | 24 |
| **Federal Agencies** |  | 1 | 1 | 0 | 3 | 0 | 4 | 2 | 0 | 3 | 2 | 0 | 0 | 0 | 3 | 1 | **20** | 5 |
| **RHBs** | 0 | 4 | 16 | 0 | 12 | 0 | 32 | 0 | 0 | 32 | 16 | 0 | 0 | 48 | 0 | 0 | **160** | 14.5 |
| **ZHDs** | 18 | 1 | 5 | 0 | 9 | 2 | 1 | 0 | 0 | 6 | 5 | 0 | 0 | 1 | 0 | 2 | **50** | 3 |
| **District health offices** | 19 | 2 | 1 | 0 | 0 | 2 | 2 | 0 | 5 | 10 |  | 0 | 0 | 0 | 0 | 4 | **45** | 2 |
| **Specialized/Ref Hospital** | 37 | 3 | 6 | 0 | 1 | 24 | 0 | 0 | 0 | 5 | 2 | 0 | 0 | 5 | 0 | 16 | **99** | 20 |
| **General Hospital** | 28 | 4 | 1 | 0 | 1 | 27 | 1 | 1 | 0 | 22 | 2 | 2 | 2 | 4 | 1 | 2 | **98** | 8 |
| **Primary Hospital** | 20 | 3 | 4 | 0 | 0 | 6 | 0 | 0 | 1 | 2 | 0 | 1 | 0 | 0 | 0 | 6 | **43** | 5 |
| **Health Center** | 19 | 2 | 0 | 0 | 0 | 2 | 0 | 0 | 2 | 0 | 0 | 1 | 0 | 0 | 0 | 0 | **26** | 1 |
| **Total** | **141** | **21** | **37** | **0** | **30** | **63** | **40** | **3** | **8** | **82** | **31** | **4** | **2** | **62** | **6** | **35** | **565** |  |

Table 3: Turnover of HIS manpower at each level of the health system

| **Type of organization** | **Health Informatics** | | | | **M & E** | | **Information Technology** | | | | **Health science** | | | | **Statistics** | | | **Computer Science** | | | **Others** | **Total** |
| --- | --- | --- | --- | --- | --- | --- | --- | --- | --- | --- | --- | --- | --- | --- | --- | --- | --- | --- | --- | --- | --- | --- |
|  | **Diploma** | **BSc** | **Masters** | **PhD** | **Masters** | **PhD** | **Diploma** | **BSc** | **Masters** | **PhD** | **Diploma** | **BSc** | **Masters** | **PhD** | **BSc** | **Masters** | **PhD** | **BSc** | **Masters** | **PhD** |  |  |
| **MOH** | 0 | 3 | 1 | 0 | 2 | 0 | 0 | 2 | 0 | 0 | 0 | 3 | 1 | 0 | 3 | 0 | 0 | 3 | 2 | 0 | 3 | 23 |
| **Federal Agencies** | 0 | 0 | 0 | 0 | 2 | 0 | 2 | 2 | 1 | 0 | 0 | 0 | 0 | 0 | 0 | 2 | 0 | 1 | 1 | 1 | 0 | 12 |
| **RHBs** | 5 | 0 | 1 | 0 | 9 | 0 | 4 | 6 | 0 | 0 | 0 | 17 | 19 | 1 | 3 | 0 | 0 | 5 | 0 | 0 | 0 | 70 |
| **ZHDs** | 10 | 3 | 3 | 0 | 1 | 0 | 3 | 0 | 0 | 0 | 1 | 2 | 0 | 0 | 0 | 0 | 0 | 1 | 0 | 0 | 3 | 27 |
| **District health offices** | 6 | 0 | 0 | 0 | 0 | 0 | 0 | 0 | 0 | 0 | 3 | 3 | 0 | 0 | 0 | 0 | 0 | 0 | 0 | 0 | 1 | 13 |
| **Specialized/Ref Hospital** | 3 | 1 | 3 | 0 | 0 | 0 | 0 | 1 | 0 | 0 | 1 | 0 | 2 | 0 | 0 | 0 | 0 | 0 | 0 | 0 | 3 | 14 |
| **General Hospital** | 9 | 0 | 0 | 0 | 2 | 0 | 0 | 0 | 0 | 0 | 4 | 0 | 0 | 0 | 3 | 1 | 0 | 0 | 0 | 0 | 0 | 19 |
| **Primary Hospital** | 8 | 1 | 3 | 0 | 0 | 0 | 0 | 0 | 0 | 0 | 2 | 3 | 1 | 0 | 1 | 0 | 0 | 3 | 4 | 0 | 2 | 28 |
| **Health Center** | 7 | 6 | 2 | 0 | 1 | 0 | 0 | 3 | 0 | 0 | 1 | 0 | 0 | 0 | 3 | 5 | 0 | 2 | 4 | 0 | 4 | 38 |
| **Total** | **48** | **14** | **13** | **0** | **17** | **0** | **9** | **14** | **1** | **0** | **12** | **28** | **23** | **1** | **13** | **8** | **0** | **15** | **11** | **1** | **16** | **244** |

Table 4: Experts’ rating for criterions used for human resource forecasting for HIS in Ethiopia

| **#** | **criterions** | **Expert-1** | **Expert-2** | **Expert-3** | **Expert-4** | **Expert-5** | **Expert-6** | **Expert-7** | **Expert-8** | **Expert-9** | **Expert-10** | **Expert-11** | **Expert-12** | **Expert-13** | **Overall Weight (100%)** |
| --- | --- | --- | --- | --- | --- | --- | --- | --- | --- | --- | --- | --- | --- | --- | --- |
|  | Expansion plan | 30 | 18 | 8 | 8 | 12 | 20 | 20 | 5 | 5 | 7 | 39 | 20 | 8 | 15.4 |
|  | Current number of HR for HIS | 15 | 9 | 10 | 8 | 9 | 8 | 8 | 40 | 20 | 4 | 10 | 8 | 8 | 12.1 |
|  | Current number of organizations | 5 | 9 | 8 | 10 | 9 | 8 | 8 | 30 | 3 | 10 | 15 | 10 | 8 | 10.2 |
|  | Strategic plans | 15 | 14 | 7 | 7 | 10 | 0 | 0 | 3 | 10 | 11 | 5 | 14 | 8 | 8.0 |
|  | eHealth initiatives | 0 | 8 | 7 | 7 | 10 | 5 | 5 | 1 | 14 | 20 | 4 | 12 | 7 | 7.7 |
|  | No. of standard HIS positions | 5 | 9 | 9 | 8 | 9 | 8 | 8 | 10 | 6 | 5 | 3 | 8 | 8 | 7.4 |
|  | Budget for health | 5 | 2 | 6 | 7 | 5 | 10 | 10 | 2 | 10 | 12 | 6 | 4 | 8 | 6.7 |
|  | HR Turnover/attrition rate | 0 | 2 | 8 | 8 | 8 | 15 | 15 | 0.5 | 9 | 6 | 0.5 | 7 | 6 | 6.5 |
|  | 5 years trend of HR for HIS | 10 | 5 | 6 | 7 | 7 | 10 | 10 | 4 | 3 | 4 | 2 | 3 | 7 | 6.0 |
|  | Work load | 5 | 8 | 9 | 6 | 11 | 5 | 5 | 1 | 2 | 10 | 3 | 6 | 7 | 6.0 |
|  | Higher education capacity | 5 | 5 | 6 | 7 | 3 | 3 | 3 | 1 | 8 | 3 | 3 | 3 | 6 | 4.3 |
|  | Population growth rate | 5 | 5 | 5 | 8 | 4 | 4 | 4 | 1 | 3 | 2 | 3 | 2 | 6 | 4.0 |
|  | Professional mix | 0 | 5 | 8 | 5 | 2 | 1 | 1 | 0.5 | 2 | 5 | 6 | 2 | 7 | 3.4 |
|  | Retirement/Death | 0 | 1 | 3 | 4 | 1 | 3 | 3 | 1 | 5 | 1 | 0.5 | 1 | 6 | 2.3 |
|  | Total | 100 | 100 | 100 | 100 | 100 | 100 | 100 | 100 | 100 | 100 | 100 | 100 | 100 | 100 |

*Each expert rated the contribution of all factors in HR forecasting out of 100%.*

Table 5: Total estimated HIS workforce need disaggregated by Organizational level in Ethiopia from 2021 to 2030.

| **Organization** | **Total Current Staff (2020)** | **Total Estimated HR for HIS from 2021 to 2030** | | | | | | | | | |
| --- | --- | --- | --- | --- | --- | --- | --- | --- | --- | --- | --- |
|  |  | 2021 | 2022 | 2023 | 2024 | 2025 | 2026 | 2027 | 2028 | 2029 | 2030 |
| MOH | 24 | 32 | 38 | 47 | 51 | 52 | 59 | 64 | 73 | 82 | 90 |
| Federal agencies | 84 | 84 | 84 | 84 | 98 | 91 | 98 | 91 | 112 | 105 | 105 |
| Regional Health Bureaus | 160 | 164 | 170 | 173 | 184 | 199 | 219 | 241 | 262 | 273 | 276 |
| Regional agencies* | - | 31 | 42 | 58 | 58 | 60 | 60 | 62 | 62 | 63 | 63 |
| Zonal Health Department | 172 | 230 | 293 | 336 | 352 | 407 | 456 | 511 | 526 | 576 | 624 |
| District Health Office | 1428 | 1803 | 1943 | 1817 | 2182 | 2153 | 2353 | 2488 | 2688 | 2789 | 3060 |
| Specialized/Referral Hospitals | 857 | 1011 | 1011 | 965 | 1150 | 1150 | 1110 | 1274 | 1274 | 1240 | 1419 |
| General Hospitals | 945 | 1274 | 1274 | 1329 | 1636 | 1646 | 1646 | 1958 | 1908 | 1918 | 2100 |
| Primary Hospitals | 2535 | 3029 | 3029 | 3029 | 3530 | 3530 | 3530 | 4027 | 4027 | 4027 | 4545 |
| Health Centers | 4139 | 9972 | 9972 | 9972 | 15820 | 15820 | 15820 | 21768 | 21768 | 21768 | 27516 |
| Private Health Facilities* | - | 1500 | 2000 | 2500 | 5000 | 6000 | 7000 | 8000 | 9000 | 10000 | 10000 |
| Universities* | - | 82 | 82 | 164 | 164 | 266 | 266 | 368 | 368 | 480 | 480 |
| Health Science Colleges* | - | 76 | 76 | 152 | 152 | 228 | 228 | 304 | 304 | 378 | 378 |
| **National** | **10344** | **19281** | **20003** | **20610** | **30377** | **31600** | **32845** | **41154** | **42372** | **43698** | **50,656** |

*Indicates baseline data for the current status were not available, and estimation was done using the current number of organizations and domain expert opinion

Table 6: Human Resource Need for HIS disaggregated by profession and level of education in Ethiopia from 2021 to 2030.

| **Profession** | **Level of education** | **Total Current Staff (2011)** | **Total Estimated HR for HIS from 2021 to 2030** | | | | | | | | | |
| --- | --- | --- | --- | --- | --- | --- | --- | --- | --- | --- | --- | --- |
|  |  |  | 2021 | 2022 | 2023 | 2024 | 2025 | 2026 | 2027 | 2028 | 2029 | 2030 |
| **Health Informatics** | **Diploma** | 5685 | 11183 | 11464 | 11499 | 17457 | 17766 | 18266 | 23361 | 23840 | 24245 | 28646 |
|  | **BSc** | 857 | 2181 | 2252 | 2322 | 3647 | 3781 | 3891 | 5206 | 5316 | 5453 | 6761 |
|  | **Masters** | 364 | 783 | 810 | 934 | 1264 | 1346 | 1365 | 1744 | 1777 | 1855 | 2194 |
|  | **PhD** | 0 | 8 | 8 | 17 | 17 | 25 | 26 | 45 | 45 | 64 | 64 |
| **IT/computer science** | **Diploma** | 1542 | 3161 | 3370 | 3581 | 5711 | 6210 | 6672 | 8114 | 8556 | 9048 | 9936 |
|  | **BSc** | 265 | 545 | 643 | 731 | 1012 | 1154 | 1282 | 1592 | 1724 | 1862 | 2169 |
|  | **Masters** | 48 | 95 | 109 | 151 | 169 | 203 | 215 | 270 | 275 | 309 | 317 |
|  | **PhD** | 0 | 4 | 4 | 8 | 9 | 13 | 13 | 17 | 17 | 21 | 21 |
| **M and E** | **Masters** | 98 | 129 | 141 | 161 | 193 | 205 | 221 | 248 | 266 | 282 | 308 |
|  | **PhD** | 0 | 0 | 0 | 0 | 0 | 0 | 0 | 0 | 1 | 1 | 1 |
| **Statistics/ Biostatistics** | **BSc** | 34 | 48 | 57 | 64 | 80 | 80 | 75 | 83 | 83 | 83 | 97 |
|  | **Masters** | 32 | 33 | 45 | 61 | 71 | 81 | 93 | 93 | 103 | 119 | 119 |
|  | **PhD** | 0 | 0 | 0 | 0 | 0 | 0 | 0 | 0 | 0 | 1 | 1 |
| **Others** | | 1419 | 1111 | 1100 | 1081 | 747 | 736 | 726 | 381 | 369 | 355 | 22 |
| **Total** | | **10344** | **19281** | **20003** | **20610** | **30377** | **31600** | **32845** | **41154** | **42372** | **43698** | **50656** |

Table 7: Human resource need for HIS by MOH from 2020 to 2030

| **Profession** | **Level of education**  **n** | **Total Current Staff (2020)** | **Total Estimated HR for HIS from 2021 to 2030** | | | | | | | | | |  |
| --- | --- | --- | --- | --- | --- | --- | --- | --- | --- | --- | --- | --- | --- |
|  |  |  | 2021 | 2022 | 2023 | 2024 | 2025 | 2026 | 2027 | 2028 | 2029 | 2030 | |
| **Health Informatics** | **BSc** | 1 | 2 | 3 | 1 | 2 | 0 | 0 | 0 | 0 | 0 | 0 | |
|  | **Masters** | 3 | 5 | 7 | 12 | 14 | 14 | 16 | 18 | 22 | 24 | 27 | |
|  | **PhD** | 0 | 0 | 0 | 1 | 1 | 1 | 2 | 2 | 2 | 3 | 3 | |
| **IT/computer science** | **BSc** | 4 | 4 | 2 | 2 | 0 | 0 | 0 | 0 | 0 | 0 | 0 | |
|  | **Masters** | 2 | 4 | 8 | 10 | 12 | 12 | 14 | 15 | 17 | 19 | 21 | |
|  | **PhD** | 0 | 0 | 0 | 0 | 1 | 1 | 1 | 1 | 1 | 1 | 1 | |
| **M and E** | **Masters** | 4 | 6 | 6 | 8 | 9 | 10 | 12 | 14 | 16 | 18 | 22 | |
|  | **PhD** | 0 | 0 | 0 | 0 | 0 | 0 | 0 | 0 | 1 | 1 | 1 | |
| **Statistics/ Biostatistics** | **Masters** | 0 | 1 | 4 | 5 | 6 | 8 | 10 | 10 | 12 | 13 | 14 | |
|  | **PhD** | 0 | 0 | 0 | 0 | 0 | 0 | 0 | 0 | 0 | 1 | 1 | |
| **Others** | | 10 | 10 | 8 | 8 | 6 | 6 | 4 | 4 | 2 | 2 | 0 | |
| **Total** | | **24** | **32** | **38** | **47** | **51** | **52** | **59** | **64** | **73** | **82** | **90** | |

Table 8: Human resource need for HIS by federal agencies from 2020 to 2030

| **Profession** | **Level of education** | **Total Current**  **Staff (2020)** | **Total Estimated HR for HIS from 2021 to 2030** | | | | | | | | | |
| --- | --- | --- | --- | --- | --- | --- | --- | --- | --- | --- | --- | --- |
|  |  |  | 2021 | 2022 | 2023 | 2024 | 2025 | 2026 | 2027 | 2028 | 2029 | 2030 |
| **Health Informatics** | **BSc** | 7 | 7 | 7 | 7 | 7 | 7 | 14 | 7 | 14 | 14 | 14 |
|  | **Masters** | 0 | 0 | 7 | 7 | 7 | 14 | 14 | 21 | 28 | 28 | 28 |
| **IT/computer science** | **BSc** | 21 | 21 | 21 | 14 | 14 | 7 | 7 | 7 | 14 | 14 | 14 |
|  | **Masters** | 7 | 7 | 7 | 14 | 14 | 14 | 14 | 21 | 21 | 21 | 21 |
| **M&E** | **Masters** | 7 | 7 | 7 | 7 | 14 | 14 | 14 | 14 | 14 | 14 | 14 |
| **Statistics/ Biostatistics** | **BSc** | 0 | 0 | 0 | 7 | 7 | 7 | 7 | 0 | 0 | 0 | 0 |
|  | **Masters** | 0 | 0 | 0 | 0 | 7 | 7 | 7 | 7 | 7 | 14 | 14 |
| **Others** | | 42 | 42 | 35 | 28 | 28 | 21 | 21 | 14 | 14 | 0 | 0 |
| **Total** | | **84** | **84** | **84** | **84** | **98** | **91** | **98** | **91** | **112** | **105** | **105** |

***Agencies*****- EPHI, FMHACA, HAPCO, PFSA, Blood Bank, AHRI, Health Insurance*

Table9: Human resource need for HIS by Health Bureaus from 2020 to 2030

|  |  | **Total Current Staff (2020)** |  | | | | | | | | | |
| --- | --- | --- | --- | --- | --- | --- | --- | --- | --- | --- | --- | --- |
|  |  |  | **Total Estimated HR for HIS from 2021 to 2030** | | | | | | | | | |
| **Profession** | **Level of education** |  | 2021 | 2022 | 2023 | 2024 | 2025 | 2026 | 2027 | 2028 | 2029 | 2030 |
| **Health Informatics** | **Diploma** | 11 | 9 | 11 | 10 | 10 | 9 | 9 | 9 | 11 | 11 | 11 |
|  | **BSc** | 0 | 2 | 2 | 5 | 5 | 15 | 20 | 25 | 30 | 32 | 32 |
|  | **Masters** | 18 | 20 | 25 | 28 | 32 | 36 | 40 | 45 | 50 | 55 | 55 |
|  | **PhD** | 0 | 0 | 0 | 0 | 0 | 0 | 0 | 11 | 11 | 11 | 11 |
| **IT/computer science** | **Diploma** | 18 | 18 | 14 | 18 | 18 | 18 | 20 | 20 | 20 | 20 | 20 |
|  | **BSc** | 24 | 26 | 24 | 28 | 36 | 41 | 46 | 51 | 53 | 53 | 53 |
|  | **Masters** | 2 | 2 | 5 | 7 | 9 | 12 | 15 | 17 | 20 | 22 | 22 |
| **M and E** | **Masters** | 17 | 17 | 18 | 18 | 19 | 19 | 22 | 23 | 25 | 27 | 28 |
| **Statistics/ Biostatistics** | **BSc** | 4 | 4 | 6 | 6 | 8 | 8 | 10 | 11 | 11 | 11 | 11 |
|  | **Masters** | 2 | 2 | 3 | 3 | 5 | 5 | 7 | 7 | 9 | 9 | 11 |
| **Others** | | 64 | 64 | 62 | 50 | 42 | 36 | 30 | 22 | 22 | 22 | 22 |
| **Total** | | **160** | **164** | **170** | **173** | **184** | **199** | **219** | **241** | **262** | **273** | **276** |

Table 10: Human resource need for HIS by regional agencies from 2020 to 2030

| **Profession** | **Level of education** | **Total Estimated HR for HIS from 2021 to 2030** | | | | | | | | | |
| --- | --- | --- | --- | --- | --- | --- | --- | --- | --- | --- | --- |
|  |  | 2021 | 2022 | 2023 | 2024 | 2025 | 2026 | 2027 | 2028 | 2029 | 2030 |
| **Health Informatics** | **BSc** | 7 | 7 | 7 | 7 | 7 | 7 | 7 | 7 | 7 | 7 |
|  | **Masters** | 7 | 7 | 9 | 9 | 11 | 11 | 13 | 13 | 14 | 14 |
| **IT/computer science** | **BSc** | 3 | 0 | 0 | 0 | 0 | 0 | 0 | 0 | 0 | 0 |
|  | **Masters** | 7 | 14 | 14 | 14 | 14 | 21 | 21 | 21 | 21 | 21 |
| **M and E** | **Masters** | 7 | 7 | 14 | 14 | 14 | 14 | 14 | 14 | 14 | 14 |
| **Statistics/ Biostatistics** | **BSc** | 0 | 7 | 7 | 7 | 7 | 0 | 0 | 0 | 0 | 0 |
|  | **Masters** | 0 | 0 | 7 | 7 | 7 | 7 | 7 | 7 | 7 | 7 |
| **Total** | | **31** | **42** | **58** | **58** | **60** | **60** | **62** | **62** | **63** | **63** |

***Agencies*****- Regional EPHI, FMHACA, HAPCO, PFSA, Blood Bank, AHRI, Health Insurance*

Table 11: Human resource need for HIS by Zonal Health Departments from 2020 to 2030

| **Profession** | **Level of education** | **Total Current Staff (2020)** | **Total Estimated HR for HIS from 2021 to 2030** | | | | | | | | | |
| --- | --- | --- | --- | --- | --- | --- | --- | --- | --- | --- | --- | --- |
|  |  |  | 2021 | 2022 | 2023 | 2024 | 2025 | 2026 | 2027 | 2028 | 2029 | 2030 |
| **Health Informatics** | **Diploma** | 78 | 78 | 78 | 78 | 39 | 39 | 39 | 39 | 0 | 0 | 0 |
|  | **BSc** | 8 | 8 | 8 | 4 | 4 | 4 | 2 | 2 | 0 | 0 | 0 |
|  | **Masters** | 23 | 39 | 52 | 65 | 78 | 91 | 104 | 117 | 134 | 147 | 156 |
| **IT/computer science** | **Diploma** | 8 | 8 | 8 | 4 | 4 | 4 | 2 | 2 | 0 | 0 | 0 |
|  | **BSc** | 8 | 31 | 62 | 85 | 108 | 131 | 154 | 177 | 200 | 217 | 234 |
| **M and E** | **Masters** | 39 | 50 | 61 | 72 | 83 | 94 | 105 | 116 | 130 | 142 | 156 |
| **Bio/statistics** | **Masters** | 0 | 8 | 16 | 24 | 32 | 40 | 48 | 56 | 62 | 70 | 78 |
| **Others** | | 8 | 8 | 8 | 4 | 4 | 4 | 2 | 2 | 0 | 0 | 0 |
| **Total** | | **172** | **230** | **293** | **336** | **352** | **407** | **456** | **511** | **526** | **576** | **624** |

Table 12: Human resource need for HIS by District Health offices from 2020 to 2030

| **Profession** | **Level of education** | **Total Current Staff (2020)** | **Total Estimated HR for HIS from 2021 to 2030** | | | | | | | | | |
| --- | --- | --- | --- | --- | --- | --- | --- | --- | --- | --- | --- | --- |
|  |  |  | 2021 | 2022 | 2023 | 2024 | 2025 | 2026 | 2027 | 2028 | 2029 | 2030 |
| **Health Informatics** | **Diploma** | 918 | 918 | 918 | 688 | 688 | 459 | 459 | 229 | 229 | 114 | 0 |
|  | **BSc** | 102 | 180 | 250 | 302 | 402 | 502 | 602 | 702 | 802 | 910 | 1020 |
|  | **Masters** | 0 | 255 | 255 | 255 | 510 | 510 | 510 | 765 | 765 | 765 | 1020 |
| **IT/computer science** | **Diploma** | 102 | 90 | 90 | 90 | 60 | 60 | 60 | 30 | 30 | 30 | 0 |
|  | **BSc** | 102 | 180 | 250 | 302 | 402 | 502 | 602 | 702 | 802 | 910 | 1020 |
| **Others** | | 204 | 180 | 180 | 180 | 120 | 120 | 120 | 60 | 60 | 60 | 0 |
| **Total** | | **1428** | **1803** | **1943** | **1817** | **2182** | **2153** | **2353** | **2488** | **2688** | **2789** | **3060** |

Table 13: Human resource need for HIS by Specialized /Referral Hospitals from 2020 to 2030.

| **Profession** | **Level of education** | **Average Current**  **Staff per CSH** | **Total Current Staff (2020)** | **Total Estimated HR for HIS from 2021 to 2030** | | | | | | | | | |
| --- | --- | --- | --- | --- | --- | --- | --- | --- | --- | --- | --- | --- | --- |
|  |  |  |  | 2021 | 2022 | 2023 | 2024 | 2025 | 2026 | 2027 | 2028 | 2029 | 2030 |
| **Health Informatics** | **Diploma** | 7.4 | 318 | 442 | 442 | 442 | 566 | 566 | 566 | 690 | 690 | 690 | 817 |
|  | **BSc** | 0.6 | 26 | 62 | 62 | 62 | 100 | 100 | 100 | 138 | 138 | 138 | 172 |
|  | **Masters** | 1.2 | 52 | 35 | 35 | 35 | 28 | 28 | 28 | 9 | 9 | 9 | 0 |
| **IT/computer science** | **Diploma** | 4.8 | 206 | 206 | 206 | 160 | 160 | 160 | 120 | 120 | 120 | 86 | 86 |
|  | **BSc** | 1 | 43 | 53 | 53 | 53 | 63 | 63 | 63 | 73 | 73 | 73 | 86 |
|  | **Masters** | 0 | 0 | 22 | 22 | 22 | 44 | 44 | 44 | 66 | 66 | 66 | 86 |
| **M and E** | **Masters** | 0.2 | 9 | 29 | 29 | 29 | 45 | 45 | 45 | 62 | 62 | 62 | 86 |
| **Bio/statistics** | **BSc** | 0 | 0 | 22 | 22 | 22 | 44 | 44 | 44 | 66 | 66 | 66 | 86 |
| **Others** | | 4.6 | 198 | 140 | 140 | 140 | 100 | 100 | 100 | 50 | 50 | 50 | 0 |
| **Total** | | **19.4** | **852** | **1011** | **1011** | **965** | **1150** | **1150** | **1110** | **1274** | **1274** | **1240** | **1419** |

Table 14: Human resource need for HIS by General Hospitals from 2020 to 2030

| **Profession** | **Level of education** | **Total Current Staff (2020)** | **Total Estimated HR for HIS from 2021 to 2030** | | | | | | | | | |
| --- | --- | --- | --- | --- | --- | --- | --- | --- | --- | --- | --- | --- |
|  |  |  | 2021 | 2022 | 2023 | 2024 | 2025 | 2026 | 2027 | 2028 | 2029 | 2030 |
| **Health Informatics** | **Diploma** | 345 | 645 | 645 | 645 | 945 | 945 | 945 | 1200 | 1200 | 1200 | 1350 |
|  | **BSc** | 45 | 125 | 125 | 125 | 205 | 205 | 205 | 250 | 250 | 250 | 300 |
|  | **Masters** | 15 | 60 | 60 | 115 | 115 | 125 | 125 | 140 | 140 | 150 | 150 |
| **IT/computer science** | **Diploma** | 345 | 280 | 280 | 280 | 215 | 215 | 215 | 215 | 165 | 165 | 150 |
|  | **BSc** | 60 | 85 | 85 | 85 | 105 | 105 | 105 | 130 | 130 | 130 | 150 |
|  | **Masters** | 30 | 22 | 22 | 22 | 14 | 14 | 14 | 6 | 6 | 6 | 0 |
| **M and E** | **Masters** | 15 | 13 | 13 | 13 | 9 | 9 | 9 | 5 | 5 | 5 | 0 |
| **Statistics/ Biostatistics** | **BSc** | 30 | 22 | 22 | 22 | 14 | 14 | 14 | 6 | 6 | 6 | 0 |
|  | **Masters** | 30 | 22 | 22 | 22 | 14 | 14 | 14 | 6 | 6 | 6 | 0 |
| **Others** | | 30 | 0 | 0 | 0 | 0 | 0 | 0 | 0 | 0 | 0 | 0 |
| **Total** | | **945** | **1274** | **1274** | **1329** | **1636** | **1646** | **1646** | **1958** | **1908** | **1918** | **2100** |

Table 15: Human resource need for HIS by Primary Hospitals from 2020 to 2030.

| **Profession** | **Level of education** | **Total Current Staff (2020))** | **Total Estimated HR for HIS from 2021 to 2030.** | | | | | | | | | | **Average HR per**  **PH after ten years** |
| --- | --- | --- | --- | --- | --- | --- | --- | --- | --- | --- | --- | --- | --- |
|  |  |  | 2021 | 2022 | 2023 | 2024 | 2025 | 2026 | 2027 | 2028 | 2029 | 2030 |  |
| **Health Informatics** | **Diploma** | 1263 | 1704 | 1704 | 1704 | 2145 | 2145 | 2145 | 2586 | 2586 | 2586 | 3030 | 6 |
|  | **BSc** | 202 | 277 | 277 | 277 | 352 | 352 | 352 | 429 | 429 | 429 | 505 | 1 |
|  | **Masters** | 253 | 316 | 316 | 316 | 379 | 379 | 379 | 432 | 432 | 432 | 505 | 1 |
| **IT/computer science** | **Diploma** | 404 | 303 | 303 | 303 | 201 | 201 | 201 | 101 | 101 | 101 | 0 | 0 |
|  | **BSc** | 0 | 126 | 126 | 126 | 252 | 252 | 252 | 378 | 378 | 378 | 505 | 1 |
| **Others** | | 404 | 303 | 303 | 303 | 201 | 201 | 201 | 101 | 101 | 101 | 0 | 0 |
| **Total** | | **2526** | **3029** | **3029** | **3029** | **3530** | **3530** | **3530** | **4027** | **4027** | **4027** | **4545** | **9** |

Table 16: Human resource need for HIS by Health Centers from 2020 to *2030*

| **Profession** | **Level of education** | **Total Current Staff (2020)** | **Total Estimated HR for HIS from 2021 to 2030 C.** | | | | | | | | | |
| --- | --- | --- | --- | --- | --- | --- | --- | --- | --- | --- | --- | --- |
|  |  |  | 2021 | 2022 | 2023 | 2024 | 2025 | 2026 | 2027 | 2028 | 2029 | 2030 |
| **Health Informatics** | **Diploma** | 2752 | 6650 | 6650 | 6650 | 10550 | 10550 | 10550 | 14550 | 14550 | 14550 | 18344 |
|  | **BSc** | 459 | 1490 | 1490 | 1490 | 2521 | 2521 | 2521 | 3552 | 3552 | 3552 | 4586 |
| **IT/computer science** | **Diploma** | 459 | 1490 | 1490 | 1490 | 2521 | 2521 | 2521 | 3552 | 3552 | 3552 | 4586 |
| **Others** | | 459 | 342 | 342 | 342 | 228 | 228 | 228 | 114 | 114 | 114 | 0 |
| **Total** | | **4129** | **9972** | **9972** | **9972** | **15820** | **15820** | **15820** | **21768** | **21768** | **21768** | **27516** |

Table 17: Human resource need for HIS by Private Health Facilities from 2020 to 2030

| **Profession** | **Level of education** | **Total Estimated HR for HIS from 2021 to 2030** | | | | | | | | | |
| --- | --- | --- | --- | --- | --- | --- | --- | --- | --- | --- | --- |
|  |  | 2021 | 2022 | 2023 | 2024 | 2025 | 2026 | 2027 | 2028 | 2029 | 2030 |
| **Health Informatics** | **Diploma** | 750 | 1000 | 1250 | 2500 | 3000 | 3500 | 4000 | 4500 | 5000 | 5000 |
| **IT/computer science** | **Diploma** | 750 | 1000 | 1250 | 2500 | 3000 | 3500 | 4000 | 4500 | 5000 | 5000 |
| **Total** | | **1500** | **2000** | **2500** | **5000** | **6000** | **7000** | **8000** | **9000** | **10000** | **10000** |

Table 18: Human resource need for HIS by Universities from 2020 to 2030

| **Profession** | **Level of education** | **Total Estimated HR for HIS from 2021 to 2030** | | | | | | | | | | **Average HR per**  **University after 10 yrs** |
| --- | --- | --- | --- | --- | --- | --- | --- | --- | --- | --- | --- | --- |
|  |  | 2021 | 2022 | 2023 | 2024 | 2025 | 2026 | 2027 | 2028 | 2029 | 2030 |  |
| **Health Informatics** | **Diploma** | 5 | 5 | 10 | 10 | 20 | 20 | 30 | 30 | 40 | 40 | 4 |
|  | **BSc** | 5 | 5 | 10 | 10 | 20 | 20 | 30 | 30 | 40 | 40 | 4 |
|  | **Masters** | 30 | 30 | 60 | 60 | 90 | 90 | 120 | 120 | 150 | 150 | 15 |
|  | **PhD** | 8 | 8 | 16 | 16 | 24 | 24 | 32 | 32 | 50 | 50 | 5 |
| **IT/computer science** | **Diploma** | 5 | 5 | 10 | 10 | 20 | 20 | 30 | 30 | 40 | 40 | 4 |
|  | **BSc** | 5 | 5 | 10 | 10 | 20 | 20 | 30 | 30 | 40 | 40 | 4 |
|  | **Masters** | 20 | 20 | 40 | 40 | 60 | 60 | 80 | 80 | 100 | 100 | 10 |
|  | **PhD** | 4 | 4 | 8 | 8 | 12 | 12 | 16 | 16 | 20 | 20 | 2 |
| **Total** | | **82** | **82** | **164** | **164** | **266** | **266** | **368** | **368** | **480** | **480** | **48** |

Table 19: Human resource need for HIS by Health Science Colleges from 2020 to 2030

| **Profession** | **Level of education** | **T Total Estimated HR for HIS from 2021 to 2030** | | | | | | | | | | **Average HR per**  **HSC after 10 yrs** |
| --- | --- | --- | --- | --- | --- | --- | --- | --- | --- | --- | --- | --- |
|  |  | 2021 | 2022 | 2023 | 2024 | 2025 | 2026 | 2027 | 2028 | 2029 | 2030 |  |
| **Health Informatics** | **Diploma** | 11 | 11 | 22 | 22 | 33 | 33 | 44 | 44 | 54 | 54 | 2 |
|  | **BSc** | 16 | 16 | 32 | 32 | 48 | 48 | 64 | 64 | 81 | 81 | 3 |
|  | **Masters** | 16 | 16 | 32 | 32 | 48 | 48 | 64 | 64 | 81 | 81 | 3 |
| **IT/computer science** | **Diploma** | 11 | 11 | 22 | 22 | 33 | 33 | 44 | 44 | 54 | 54 | 2 |
|  | **BSc** | 11 | 11 | 22 | 22 | 33 | 33 | 44 | 44 | 54 | 54 | 2 |
|  | **Masters** | 11 | 11 | 22 | 22 | 33 | 33 | 44 | 44 | 54 | 54 | 2 |
| **Total** | | **76** | **76** | **152** | **152** | **228** | **228** | **304** | **304** | **378** | **378** | **12** |
